# Dispersion Mechanisms of Lignosulfonates in Concentrated TiO_2_ Suspensions and Pastes: The Effects of Molecular Weight Distributions and Ionic Composition

**DOI:** 10.3390/polym18020270

**Published:** 2026-01-20

**Authors:** Veslemøy Margrethe Selvik, Carlos Salas-Bringas, Gisle Øye

**Affiliations:** 1 Borregaard ASA, 1701 Sarpsborg, Norway; veslemoy.selvik@borregaard.com (V.M.S.); carlos.salas.bringas@borregaard.com (C.S.-B.); 2 Ugelstad Laboratory, Department of Chemical Engineering, Norwegian University of Science and Technology (NTNU), 7491 Trondheim, Norway

**Keywords:** adsorption, dispersant, lignosulfonate, rheology, suspension

## Abstract

This study investigates how molecular weight, ionic strength, and ionic composition influence the performance of sodium lignosulfonate as a dispersant for titanium dioxide (TiO_2_) suspensions. Adsorption behavior was quantified using a quartz crystal microbalance with dissipation monitoring (QCM-D), while dispersion efficiency was assessed in concentrated suspensions via particle analysis (LUMiSizer) and in pastes through rheological measurements. In salt-free conditions, no adsorption occurs; however, the observed low particle size and viscosity can be attributed to depletion stabilization by non-adsorbing lignosulfonates. Both low- and high-molecular-weight fractions exhibit dispersing performance, but high-molecular-weight lignosulfonate provides the greatest stability across electrolyte variations. Increasing ionic strength enhances adsorption, leading to elastic particle network formation and higher viscosity due to reduced Debye length. With divalent ions, this effect is stronger and promoted by divalent cation bridging. These findings underscore the importance of tailoring lignosulfonate molecular weight and dosage to operating conditions, supporting formulation strategies for mineral-rich suspensions and industrial effluents. Future work should address long-term stability, temperature effects, and behavior on hydrophobic surfaces.

## 1. Introduction

Lignosulfonates are bio-based macromolecules extracted from wood through the sulfite pulping process. The molecular structure consists of hydrophobic aromatic rings and hydrophilic functional groups such as sulfonate, carboxylate, and phenolic hydroxyl groups [1,2]. It has been reported that the degree of sulfonation decreases with increasing molecular weight [3]. Moreover, the charge-to-size ratio is a key factor influencing molecular hydrophobicity [4] and has also been identified as governing their interfacial behavior [5]. Lignosulfonates have widespread uses as dispersants and binders in industrial processes. The desire to shift from synthetic dispersants to bio-based green products is partly driven by increased focus on sustainability and a reduction in life cycle emissions. Lignosulfonates can provide dispersion performance similar to synthetic polymers, such as poly(acrylic acid) in alumina dispersion [6] while offering a reduced carbon footprint [7,8]. Their utilization is therefore expected to grow in the coming years.

Suspensions play a critical role in numerous industrial processes, and their rheological properties can be controlled using dispersants. Adding dispersants to stabilize pigments is essential in the paint industry to achieve uniform color and stability of paints. In mineral processing, dispersants are commonly employed to reduce the viscosity of suspensions to facilitate pumping. In more concentrated suspensions, such as cement, maintaining workable, flowable suspensions without excessive addition of water is desirable. Lignosulfonates have been shown to act as effective dispersants in systems containing hydrophilic particles, such as clays [9,10] and TiO_2_ [11,12], and hydrophobic particles, such as dyes [13,14,15,16,17] and coal [18,19,20]. Typically, adsorption of lignosulfonates on the surface of solid particles improves the stability [21] by preventing agglomeration of particles through a combination of electrostatic and steric repulsion [22]. The adsorption behavior of lignosulfonate has been found to depend strongly on the pH of the suspension and the isoelectric point (IEP) of the particles. Fagerholm et al. [23] found that lignosulfonates act as non-adsorbing, free polymers in Si_3_N_4_-Y_2_O_3_ systems under alkaline conditions above the IEP of the pristine particles. The same trend was observed for suspensions of zirconia above the IEP [24]. In both cases, depletion stabilization was identified as the governing stabilization mechanism. Other authors have found that adsorption takes place at pH below or close to the IEP of alumina particles [6,25].

Lignosulfonates, as opposed to synthetic dispersants, do not have regular repeating chemical structures, and they possess polydispersity in both functionalities and molecular weights [4]. This influences their interfacial activity and thereby their dispersing and binding behavior. Polydispersity also makes it necessary to consider lignosulfonates as statistical entities, which makes the interpretation of data and gaining a fundamental understanding challenging. This complicates the translation of findings into practical applications. A deeper understanding of the structure and behavior of lignosulfonate fractions with narrow distributions of molecular weights can be one way of mitigating this lack of knowledge.

The dispersive properties of lignosulfonate fractions have been reported in previous studies. Yang et al. showed that fractionated lignosulfonates performed better than unfractionated ones in dispersing dye particles [15]. Zhou et al. demonstrated that high-molecular-weight fractions of lignosulfonates resulted in high adsorption and compact films on coal particles, resulting in reduced viscosity of coal–water slurries [20]. Similarly, Li et al. reported that adsorption of lignosulfonates onto hydrophobic particles, used as active ingredients in agricultural chemicals, increased with increasing molecular weights [26]. Furthermore, Yang et al. showed that a high-molecular-weight lignosulfonate fraction adsorbed most onto titanium dioxide particles and provided the best dispersive properties [11].

Understanding the adsorption of lignosulfonates onto particles is central to understanding their dispersive properties. The quartz crystal microbalance with dissipation (QCM-D) method provides fast and direct measurements of adsorption onto solid surfaces. This technique has typically been used to study polydisperse lignosulfonates in solutions with monovalent salts. Adsorption of lignosulfonates on dye-coated surfaces increased with increasing ionic strength and was attributed to shielding of electrostatic repulsions between sulfonic groups [13]. The adsorption of lignosulfonates on oxide surfaces increased with decreasing pH [27] and formed rigid adsorption layers [25]. Li et al. found that high-molecular-weight lignosulfonate fractions with low polydispersity gave high adsorption amounts [28]. To the best of our knowledge, the influence of divalent ions on the adsorption of lignosulfonate fractions on this type of surface has not been previously reported.

The goal of this study was to improve the understanding of how different lignosulfonate fractions perform as dispersants for hydrophilic titanium dioxide (TiO_2_) particles in concentrated suspensions and pastes. The influence of ionic strength and ionic composition on the dispersion performance was examined. The adsorption of lignosulfonate fractions onto a model TiO_2_ surface was determined by QCM-D. The dispersion performance of the lignosulfonate fractions in the concentrated suspensions was evaluated from aggregate sizes measured by the LUMiSizer Dispersion Analyser (LUM GmbH, Berlin-Adlershof, Germany). The structural and flow properties of the pastes of TiO_2_ mixed with lignosulfonate fractions were determined by rheological measurements.

## 2. Materials and Method

### 2.1. Materials

Sodium lignosulfonate (DP-53329) originated from sulfite pulping of Norway spruce and was provided by Borregaard AS (Sarpsborg, Norway). The sample was supplied as 49.7% dry matter suspended in water. This was referred to as the start material (SM). Kronos 2190 rutile titanium dioxide (TiO_2_) particles were procured from Kronos Worldwide, Inc. (Dallas, TX, USA).

### 2.2. Lignosulfonate Characterization

#### 2.2.1. Fractionation

SM was diluted with deionized water to 10% dry matter and then separated into fractions using ultrafiltration. A Millipore ProScale (Merck, Burlington, MA, USA) system was used with Millipore membranes with molecular weight cut-offs of 1, 5, and 300 kDa. The filtration area was 0.1 m^2^, and the transmembrane pressure was kept between 3 and 4 MPa. The low-molecular-weight (LMW) fraction was first filtered through a 1 kDa membrane, and the retentate was then used as feed for a second filtration through a 5 kDa membrane. Diawater (addition of water to the retentate to improve filtration) was used for the second filtration, and the permeate was collected as the final fraction. The high-molecular-weight (HMW) fraction was filtered through a 300 kDa membrane, and the retentate was collected as the final fraction. The ultrafiltration scheme is shown in Supporting Information (Figure S1).

#### 2.2.2. Molecular Weight Determination

The molecular weight distributions of the lignosulfonates were determined by size exclusion chromatography as described by Fredheim et al. [3,29] using a UV detector according to Ekeberg et al. [30]. More details on the method and characterization of the fractions can be found in previous work [31].

### 2.3. Sample Preparation

#### 2.3.1. Lignosulfonate Solutions for QCM-D Measurements

Stock solutions of 1 M NaCl were prepared by dissolving NaCl in 150 mL of ultra-pure water, while stock solutions of 0.5 M CaCl_2_ were prepared by dissolving anhydrous CaCl_2_ in 100 mL of ultra-pure water. Then, 4 g L^−1^ stock solutions for each of the lignosulfonate fractions were prepared in ultra-pure water.

Prior to the experiments, appropriate amounts of stock solutions, and ultra-pure water if necessary, were mixed to make lignosulfonate solutions with ionic strengths of 0.1 M and 0.5 M, respectively. Solutions of NaCl and a mixture of NaCl and CaCl_2_ were prepared at each ionic strength, with a 6:1 Ca^2+^/Na^+^ ratio in the latter case. The lignosulfonate concentration was kept at 2 g L^−1^ in all the experiments. The highest ionic strength was below the salting-out concentration of lignosulfonates [32], and the ratio of 6:1 divalent/monovalent ions was higher than the average 3:1 ratio in Norwegian groundwater (Table S1 in Supporting Information).

#### 2.3.2. TiO_2_ Pastes for Rheological Measurements

TiO_2_ pastes were prepared by adding lignosulfonates, electrolytes, and particles into a plastic container and mixing at 2000 rpm for 5 min at ambient temperature using a Thinky ARM-310 planetary centrifugal mixer (Thinky Mixer, Tokyo, Japan).

The aim was to maximize the solid loading while still ensuring that rheological measurements could be performed using the bob-cup geometry of the rheometer. Dispersants are most critical at high solid loadings, and the aim was to push the system to extremities to better understand dispersant properties. Additionally, these concentrations are relevant in applications aiming to reduce drying costs and increase solid flux. In waterborne paints and coatings, the solid loading is typically high (50 wt% to 70 wt%) [33], so the study may also have industrial relevance beyond the fundamental scope of the study.

Initially, samples with three solid loadings were prepared without adding dispersant, resulting in different textures. To ensure that the samples were within the measurable range of the bob-cup system, a solid loading of 75 wt% was selected. Images of these pastes are shown in Supporting Information (Figure S2).

**Figure 1:**
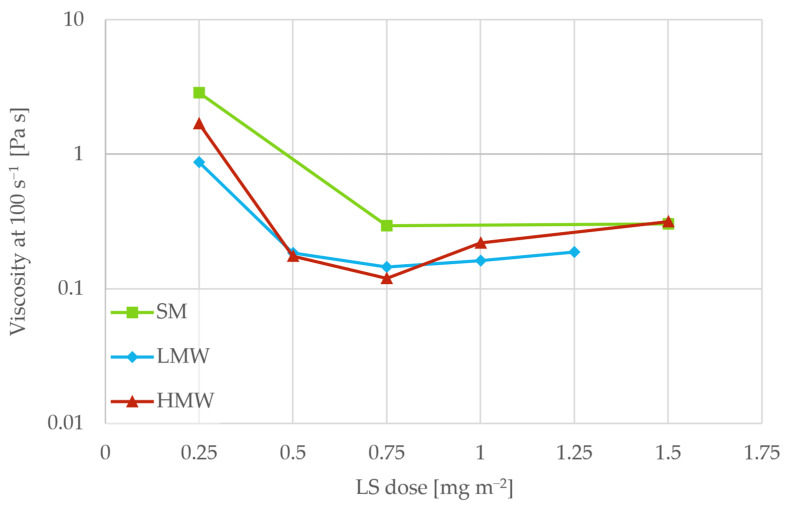
Viscosity of TiO_2_ pastes as a function of lignosulfonate dosage for the SM, LMW, and HMW fractions.

The lignosulfonate dosage was the concentration that yielded the lowest viscosity at 100 s^−1^ in the flow curves when the solid loading was 75 wt%. Figure 1 shows the viscosity for different lignosulfonate dosages as mass per surface area TiO_2_ (details on surface area measurement can be found in Supporting Information, Section S4). The lowest viscosity was observed at 0.75 mg m^−2^ for all lignosulfonate fractions, and this amount was fixed in subsequent experiments.

#### 2.3.3. Concentrated Suspensions

For particle size determination, the pastes described in Section 2.3.2 were diluted tenfold with ultra-pure water and sonicated prior to analysis. This resulted in solid and lignosulfonate loadings of 7.5 wt% and 0.75 mg m^−2^, respectively.

### 2.4. QCM-D Measurements

The adsorption of lignosulfonate fractions onto titanium dioxide-coated surfaces was measured using a Q-sense Explorer QCM-D instrument (Biolin Scientific, Gothenburg, Sweden). The atomic composition of the titanium dioxide layer on the sensor was 70.5% titanium and 29.5% oxygen (data provided by supplier). Prior to use, the sensors were first placed in a UV/ozone box for 10 min and then submerged in a 2% sodium dodecyl sulfate solution for 30 min. Then, the sensors were rinsed with ultra-pure water, dried with nitrogen gas, and exposed to UV/ozone for another 10 min. Following this treatment, a drop of ultra-pure water formed a contact angle of 23° at the surface (measured by a Teclis Tracker tensiometer (Teclis Scientific, Civrieux d’Azergues, France) at room temperature). This confirmed that any contamination was removed and that the surfaces were hydrophilic.

Adsorption measurements were performed in three stages: Baseline: A stable baseline was established by flowing the electrolyte solution corresponding to the ionic strength and composition of the subsequent lignosulfonate solution over the sensor. Adsorption: The lignosulfonate solution was continuously flushed over the sensor until adsorption equilibrium was reached (typically within 1–5 h). Washing: The system was washed with the same electrolyte solution used to establish the baseline to wash away any loosely bound material. The flow rate was maintained at 0.1 mL min^−1^ at all stages, and the temperature was kept at 20 °C in all the experiments.

The adsorbed mass was calculated from the measured frequency shifts using the Sauerbrey equation:

(1) $$\Delta m=\;-C\cdot \frac{\Delta f}{n}$$

where C is the mass sensitivity constant (17.7 ng cm^−2^ Hz^−1^ at 5 MHz), Δf is the frequency shift, and n is the harmonic number. The third harmonic (n=3) was used for all mass calculations. The Sauerbrey equation is valid for thin, rigid, and evenly distributed adsorption layers. The dissipation was low (<2 × 10^−6^) in all the measurements, which indicated rigid adsorption layers and confirmed the validity of the Sauerbrey equation. Details on data analysis can be found in Supporting Information (Figure S3). The coefficient of error on the calculated mass was found to be 10%.

### 2.5. LUMiSizer Measurements

The dispersion performance of the lignosulfonate fractions in the concentrated suspensions was determined using a LUMiSizer analytical centrifuge (LUM GmbH, Berlin-Adlershof, Germany). Then, 400 μL of suspensions were added with an autopipette to 2 mm rectangular polycarbonate cells (LUMiSizer cell, type 2). Up to 12 samples at the same time were sealed and placed horizontally with the top pointing towards the center of the centrifuge. Each suspension was studied at least once at three centrifugal speeds: 2000, 2500, and 3000 rpm at 20 °C. A light source (865 nm) was directed through the samples, and a detector measured the transmitted light along their lengths. Transmission profiles were recorded every 10 s until the instability index reached a constant value, indicating completion of phase separation. The time was typically between 20 min and 1 h, depending on the rotational speed. The “Particle Characterization” module of the LUMiSizer software (SEPView 6.4) was used to determine the velocity of the particle sedimentation front (indicated by a steep reduction in transmission) and apply Stokes law to calculate the particle sizes. Size was measured at 2000, 2500, and 3000 rpm, and the median particle size (d50, nm) was extracted from each distribution. The average of the median particle size across three centrifugal speeds and the associated standard deviation is reported.

For the suspensions without lignosulfonate present, the sedimentation was fast, and the quality of the data was therefore poor. In addition, sedimentation occurred under gravitational force, resulting in an unrepresentative sample extraction. This data was therefore not included in the graphs.

### 2.6. Rheological Measurements

A rotational rheometer, MCR102e (Anton Paar GmbH, Graz, Austria), equipped with a concentric cylinder geometry with a 27 mm diameter (CC27), was used for rheological measurements of the pastes. After loading the samples into the measurement geometry, the temperature was stabilized and maintained at 20 ± 0.05 °C for 10 min prior to pre-shearing at 150 s^−1^ for 5 min. This ensured that any structural differences caused by prior handling were minimized and provided a consistent starting state.

Amplitude sweeps (0.001–100% strain) were conducted at 10 rad s^−1^ to identify the linear viscoelastic region (LVR) and quantify storage (G′) and loss (G″) modulus under small-amplitude oscillatory shear. Preliminary tests were performed to ensure that the amplitude range and the frequency were within the torque measuring range of the instrument. The yield stress was determined as a 3% deviation in G′ from the LVR. Flow curves were obtained by increasing shear rates from 0.1 to 200 s^−1^.

To evaluate the reproducibility, flow curves were recorded in triplicate, and the average viscosity with its standard deviation was calculated at shear rates of 1, 10, and 100 s^−1^. The coefficient of variation was typically below 10% for shear rates of 10 and 100 s^−1^. Higher variability was observed at the lowest shear rate (1 s^−1^) and for the HMW fraction. The deviations observed were nevertheless smaller than the effects seen by changing conditions. Details are provided in Supporting Information (Table S2).

## 3. Results and Discussion

### 3.1. Lignosulfonate Characterization

#### 3.1.1. Molecular Weight Distributions

**Figure 2:**
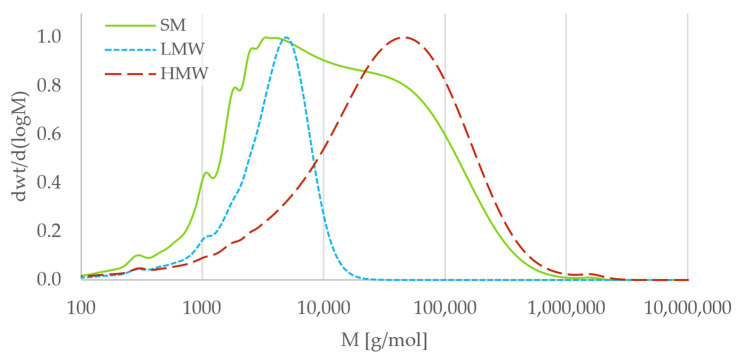
Molecular weight distributions (differential distribution) of SM, LMW, and HMW fractions. Reproduced with permission from Selvik et al. [31].

The molecular weight distributions for the fractions are shown in Figure 2.

SM had an average molecular weight of 43,500 g/mol and dispersity (Đ, M_w_/M_n_) of 13.2. The LMW had an average molecular weight of 4500 g mol^−1^ and a Đ of 2.0, while the HMW had an average molecular weight of 78,000 g mol^−1^ and a Đ of 12.0. Figure 2 shows some overlap of the LMW and HMW fractions, but the peaks are distinctly separated. The LMW fraction has a narrower distribution compared to the HMW fraction, which was attributed to the 2-step filtration used for the former.

#### 3.1.2. Chemical Characterization

Previous chemical characterization [31] showed that the bonding patterns were the same for the LMW and HMW fractions, while the functional group content (sulfonate, phenolic hydroxyl, and carboxylic acid groups) was slightly lower for the HMW fraction. Furthermore, the LMW fraction was more hydrophilic than the HMW fraction, with the unfractionated start material showing intermediate properties. The key distinction in features was the molecular weights, sizes, and hydrophobicity. Notably, the HMW fraction exhibited a more pronounced reduction in size at high ionic strength compared to the LMW fraction. The sodium content in the SM was previously found to be 4.00 mmol/g, while for the fractionated samples LMW and HMW, it was lower at 3.48 mmol/g and 2.04 mmol/g, respectively [31].

### 3.2. QCM-D Measurements of Adsorbed Mass

The pH of the electrolyte solutions was 6.4 ± 0.2, and the lignosulfonate solutions had a pH of 6.8 ± 0.2. Lignosulfonates are negatively charged in the solutions at these conditions [34]. Furthermore, the hydrophilic character of TiO_2_ stems from surface hydroxyl groups that can undergo pH-dependent dissociation. A broad range of pH values for the IEP of TiO_2_ has been reported in the literature, depending both on the crystalline structure of TiO_2_ and experimental conditions. Since the crystalline structure of the TiO_2_ coating on the sensor was not known, pH 5.8 [35] was assumed to represent the IEP (the average between typical values of anatase and rutile). Hence, it was assumed that the titanium dioxide surfaces were also negatively charged during the adsorption measurements.

The general mechanisms of adsorption of negatively charged polyelectrolytes onto negatively charged surfaces are briefly described first. The negatively charged polyelectrolytes and the surface are both surrounded by similar counterions. When the lignosulfonates approach the surface, the concentration of counterions between the polyelectrolyte and the surface increases. This causes an osmotic flow of water into the region with a higher counterion concentration that prevents the polymers from reaching the surface, forming a zone depleted of polymers close to the surface. Hence, sufficiently attractive van der Waals or hydrophobic forces are required for adsorption to occur. Increasing the ionic strength increases the adsorption, since the electrical double layers become compressed, and this allows the polymers to be closer to the surface, where the influence of the attractive forces is stronger. Furthermore, it leads to more compact polyelectrolyte conformations that allow more polymers at the surface. Furthermore, divalent ions are more effective in shielding repulsive charges and reducing the solubility of polyelectrolytes, and increased adsorption is favored [36].

Polydispersity is another aspect that influences the adsorption of polyelectrolytes. High-molecular-weight polymers have lower solubility than low-molecular-weight polymers and therefore have a higher affinity to surfaces. In solutions with polydisperse polymers, this initially leads to adsorption of the polymers with lower molecular weight (due to faster transport to the surface). These will, however, be replaced by high-molecular-weight polymers as the system approaches equilibrium.

**Figure 3:**
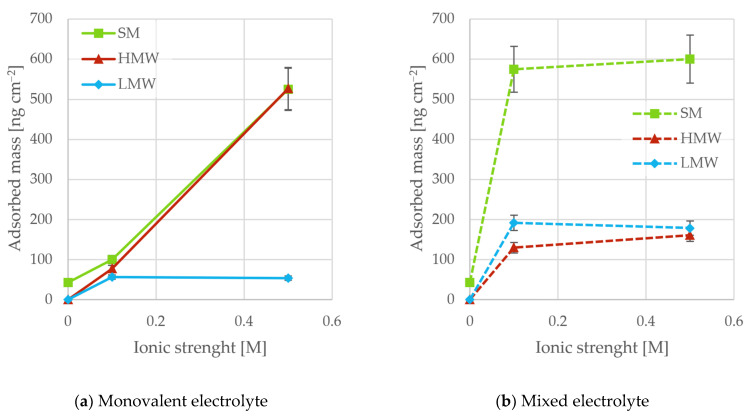
The mass adsorbed on the TiO_2_ surface for SM, LMW, and HMW fractions dissolved in solution with different ionic strengths and only monovalent salt (**a**) or a mixture of monovalent and divalent salts (**b**).

Figure 3 shows the adsorbed mass of different lignosulfonate fractions as a function of ionic strength with only monovalent counterions Figure 3a and a mixture of monovalent and divalent counterions Figure 3b.

#### 3.2.1. Effect of Molecular Weight

In the monovalent electrolyte, the SM and the HMW fractions adsorbed in higher amounts than the LMW fraction (Figure 3a). Reduced solubility and higher affinity for the surface of the high-molecular-weight polymers are possible reasons for this. Yang et al. observed a similar trend with lignosulfonate fractions originating from hardwood [11]. With divalent ions, higher adsorption was seen for the SM compared to the two fractions (Figure 3b).

#### 3.2.2. Effect of Ionic Strength

Without added salt (Figure 3a, 0 M), no adsorption was seen for the LMW or HMW fractions. In pure water, at neutral pH, both sulfonate groups and carboxylic acid groups on the lignosulfonates are ionized [5], and the TiO_2_ surface is assumed to carry a negative charge. The surface zone depleted of lignosulfonates is therefore relatively thick and out of range for van der Waals forces, promoting adsorption of lignosulfonate to the surface. The adsorption observed for SM can be due to higher concentrations of ions and impurities (from the lignosulfonates) compressing the depletion zone and allowing adsorption.

At higher ionic strengths (Figure 3a), adsorption was observed for all samples. As discussed previously, compression of the electrical double layers allows the lignosulfonates to be closer to the surface, where they come within the range of attractive forces. The Debye length is a useful representation of the extension of the electrical double layers. At 0.1 M ionic strength, it is 0.95 nm, while at 0.5 M, the length is 0.43 nm. The adsorption for SM and HMW was seen to increase accordingly. Previously, it was observed that the molecular dimensions of the HMW fraction were considerably reduced with increasing ionic strength, whereas the hydrodynamic radius of the LMW fraction showed no dependence on ionic conditions [31]. The enhanced adsorption of SM and HMW (compared to LMW) was attributed to compaction of the lignosulfonate molecule, which allowed more entities to adsorb on the surface. Increased adsorption with increasing ionic strength on hydrophilic particles is in line with work by other authors on similar materials [9,10]. For the LMW fraction, no increases in adsorption were seen, possibly due to negligible changes in molecular conformation and saturated surface adsorption.

#### 3.2.3. Effect of Ionic Composition

In the presence of calcium ions (Figure 3b), the increased adsorption (compared to only monovalent ions) was assigned to divalent cation bridging, as also reported by others [9,37]. The adsorption did not increase significantly with increased ionic strength, however. No indication of salting-out was observed, and the ionic concentrations used are below the literature values for lignosulfonates [32]. The continuous supply of lignosulfonate counteracts this effect regardless. It is therefore likely that the restricted availability of Ca^2+^ on the surface, limiting the lignosulfonate adsorption at higher ionic strengths, is the main reason for the constant adsorption at elevated ionic strength. Similar observations were made by Liu et al., who investigated the adsorption of an anionic surfactant (alcohol alkoxy sulfate) on SiO_2_ surfaces. They observed that increasing CaCl_2_ concentration enhanced adsorption only up to a saturation point, which was attributed to a finite number of surface adsorption sites [37].

### 3.3. Lignosulfonate Performance as Dispersants for TiO_2_ Particles

The presence of hydroxyl groups also gave rise to pH-dependent surface charges for the TiO_2_ particles. The IEP of the particles was found at pH 4.6 (the procedure and results for electrophoretic measurements are provided in Supporting Information (Figure S4)), which was slightly below the common range of values for rutile TiO_2_. It should be noted, however, that the surface properties (in terms of roughness and charge densities) as well as the surface curvature differed between the QCM-D sensors and the particles. Hence, the relations between the adsorption measurements and suspension behavior were only considered qualitatively.

In the absence of lignosulfonates, the particle stability against aggregation was evaluated in terms of the DLVO theory, so the floc sizes reflect a balance between repulsion due to overlapping electrical double layers and van der Waals attraction. The repulsion caused by overlapping double layers originates from an osmotic flow of water into the region with higher counterion concentration when particles approach each other, in a similar way as described for polymer adsorption above. This drives the particles in the flocs apart. Increasing ionic strength compresses the electrical double layers, allowing particles to approach more closely until attractive forces dominate over the effect of the osmotic flow.

When lignosulfonates are present, adsorption of these on the particles increases the total charge on the surface, and their stability can still partly be described by the DLVO theory. However, the adsorbed layer provides additional steric effects. If the lignosulfonates do not adsorb onto the particles, on the other hand, similar depletion effects as described earlier occur. In the following section, the dispersion performance of the lignosulfonate fractions is discussed in terms of these mechanisms.

#### 3.3.1. Concentrated Suspensions

**Figure 4:**
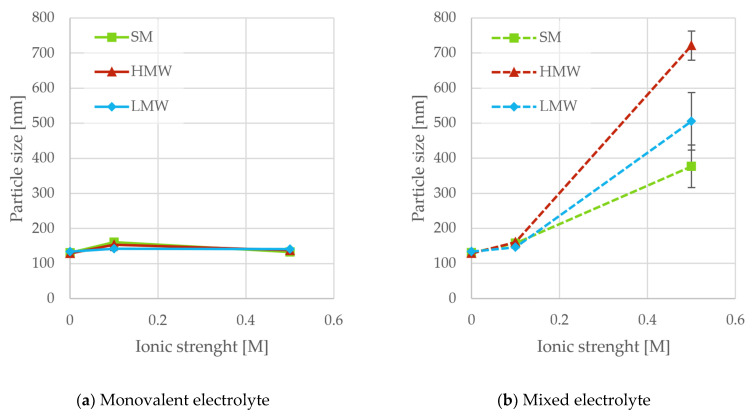
Average size of TiO_2_ as a function of ionic strength with monovalent (**a**) or a mixture of monovalent and divalent counterions (**b**).

The sizes of TiO_2_ flocs in the presence of SM, LMW, and HMW lignosulfonates are shown in Figure 4.

In the absence of a dispersant, the measured particle size in water ranged from 500 to 800 nm. However, reproducibility was limited due to rapid sedimentation and broad particle size distributions; therefore, these values should be regarded as minimum estimates. In parallel, a dry-state particle size was estimated from bulk density and BET surface as described in Supporting Information (Section S4). This approach yielded a calculated particle size of 514 nm. This calculation assumes spherical and monodisperse particles, and the results should therefore be considered a minimum value. These results provide a useful baseline for comparison with particle sizes obtained in the presence of dispersant.

Addition of lignosulfonates resulted in smaller sizes (130–160 nm, Figure 4a), independent of both lignosulfonate fraction and ionic strength. The sizes were at least a factor of 4 smaller than the flocs without lignosulfonate. This demonstrated the ability of lignosulfonates to function as dispersants. Without added electrolyte, adsorption of LMW and HMW was not detected (Figure 3a). As discussed previously, this is due to negative charges on both the particles and lignosulfonates, resulting in repulsive forces. However, a reduction in both viscosity (Figure 1) and particle size (Figure 4) compared to the samples without dispersant was observed. This suggests that lignosulfonates provide dispersion even without adsorption, which supports the inference that the mechanism is depletion stabilization. Depletion stabilization has also been reported in other studies [23,24,38]. For SM that showed some adsorption, the mechanism remains unclear. Since the ionic strength did not affect the sizes, the stability at higher ionic strengths was attributed to steric effects by the adsorbed lignosulfonates.

Figure 4b shows the particle size as a function of ionic strength with calcium ions present in the solution. The dispersion performance of all the fractions was similar to the samples with NaCl at low ionic strength (Figure 4b). At the high ionic strength, however, the dispersion performance was poorer and varied between fractions. In the QCM-D experiments, no increase in adsorption was observed with increasing ionic strength of the mixed electrolyte (Figure 3b). Since the lignosulfonate dosage remained constant across all experiments, salt-induced contraction of the lignosulfonate macromolecule (resulting in decreased surface coverage) and divalent cation-mediated bridging are the likely causes of increased aggregation at high ionic strength.

#### 3.3.2. Paste Rheology

The storage modulus (G′) and loss modulus (G″), reflecting the elastic and viscous behavior of the sample, were determined in the amplitude sweeps. The loss factor (tanδ) describes the ratio between viscous and elastic moduli. When G′ is larger than G″, the sample exhibited predominantly elastic (solid-like) behavior and tan δ values below 1. High elastic modulus (G′) is related to a higher degree of network formation and increased interparticle interactions and can therefore be used to compare dispersibility between samples. Furthermore, the sample is in the linear viscoelastic region (LVR) as long as both moduli remain constant with increasing strains. At sufficiently high stress, i.e., the yield stress, the sample begins to flow, which is indicated by a sharp decrease in G′ at the end of the LVR. Viscosity as a function of shear rate provides information on shear-thinning behavior and flow resistance, complementing the viscoelastic parameters obtained from oscillatory tests.

In this study, all samples showed solid-like behavior in the LVR and displayed shear-thinning behavior in the flow measurements. The influence of the lignosulfonate fractions, ionic strength, and ionic composition on these responses is presented in the following section. TiO_2_ pasted without lignosulfonates was used as a reference in all cases.

##### Effect of Lignosulfonate Fraction

**Figure 5:**
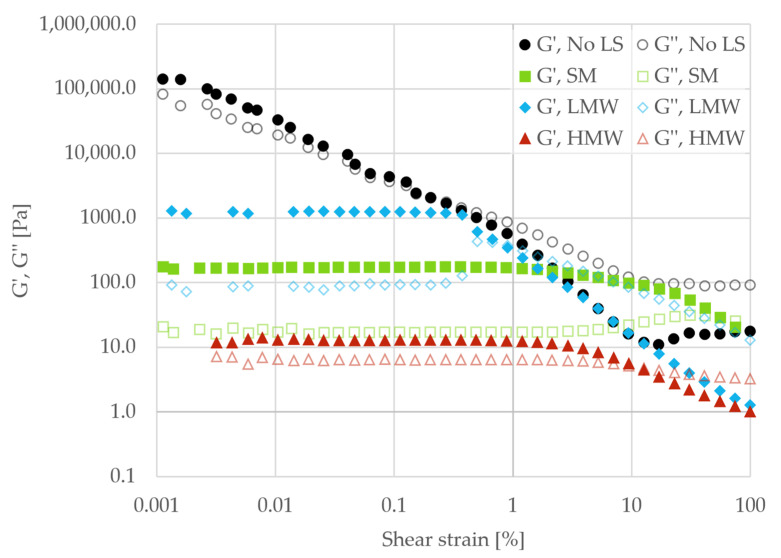
Storage (G′, filled symbols) and loss (G″, unfilled symbols) modulus as a function of strain for pastes without dispersant (no LS) and with SM, LMW, and HMW fractions as dispersants. All pastes were prepared without the addition of salt.

Figure 5 shows the strain sweep curves for the reference sample without dispersant (No LS) and the pastes with added SM, LMW, or HMW fractions.

The pastes without dispersant (no LS) had a storage modulus of 145,000 Pa and a loss factor of 0.28 at the lowest measured strain, while no LVR was observed. This indicated a fragile particle network. In the presence of dispersant, the storage modulus was generally reduced by several orders of magnitude, while broader LVRs appeared. A distinct difference was also seen between samples containing the LMW and HMW fractions. The paste with the HMW fraction had a lower storage modulus (13 Pa) and a higher loss factor (0.55) within the LVR than the paste dispersed with the LMW fraction (1223 Pa and 0.08), indicating a weakening of the particle–particle network and reduced solid-like behavior. The yield stress also decreased for the high-molecular-weight fraction (4.20 Pa for LMW and 0.15 Pa for HMW).

**Figure 6:**
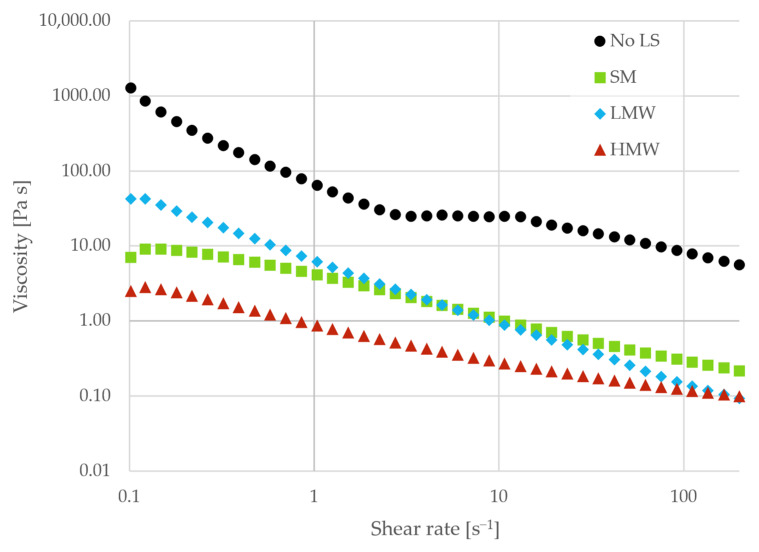
Viscosity as a function of shear rate for pastes without dispersant (No LS) and with SM, LMW, and HMW fractions as dispersants. All pastes were prepared without the addition of salt.

A similar trend was seen in the flow curves for the same samples, as shown in Figure 6. All samples showed shear-thinning behavior. The highest viscosity was seen for the bare particles (no LS), indicating a larger degree of interparticle interactions, most likely in the form of collisions under flow. A feature appeared near 10 s^−1^, which may reflect flow-induced microstructural rearrangement (e.g., formation and subsequent breakup of particle clusters) that is subsequently disrupted at higher shear rates. Upon addition of lignosulfonates, the viscosity was lowered across the entire range of shear rates, and the feature at 10 s^−1^ disappeared. The HMW fraction provided the greatest viscosity reduction. Overall, the rheological measurements showed that lignosulfonates provided dispersive effects in the samples.

##### Effect of Ionic Strength on Rheology of the Pastes

Storage modulus and loss factor in the linear viscoelastic region and yield stress for pastes with varying dispersant types under different ionic strengths can be found in Table 1.

When no lignosulfonate was present, G′ did not change appreciably with ionic strength, and no clear LVR was observed within the measured strain range. The loss factor varied between 0.43 and 0.50. As discussed previously, this indicated a fragile network.

With lignosulfonate present, the storage modulus increased with ionic strength, and the loss factor generally increased. With the high solid loading (75%) in the pastes, there were considerable connections between particles. Without salt, there were repulsive interactions between lignosulfonates and particles, resulting in more fragile structures. The addition of salt allowed adsorption of lignosulfonates, probably onto several particles simultaneously due to close packing, giving more elastic pastes.

The yield stress reached a maximum at 0.1 M NaCl. At this ionic strength, the Debye length was approximately 0.95 nm, which allowed adsorption of lignosulfonate on the surface and more elastic pastes. At 0.5 M NaCl, the Debye length was 0.43 nm, thereby lowering the electrostatic barrier to adsorption. Simultaneously, the lignosulfonate structure compacted, which yielded thinner adsorbed layers and increased interparticle connectivity. At high ionic strength, adsorbed lignosulfonate lowered the yield stress relative to the paste without lignosulfonate. This illustrates how G′ reflects small-strain network stiffness, whereas yield stress reflects the force required to break particle clusters, and that these two quantities need not correlate directly. The HMW fraction resulted in the lowest G′ at high ionic strength, indicating the greatest ionic strength tolerance.

**Figure 7:**
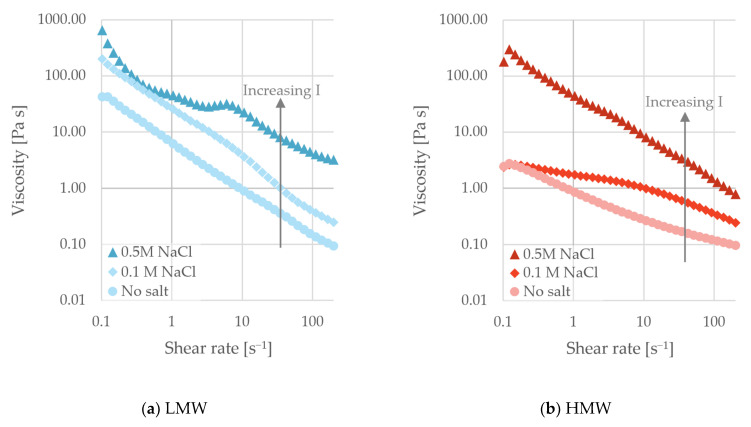
Viscosity as a function of shear rate for pastes with increasing ionic strength. The pastes were dispersed with LMW (**a**) and HMW (**b**) fractions.

Similar trends as described above were present in the flow curves for the fractions, as seen in Figure 7. The lowest viscosity was observed when no salt was added, and the values increased with ionic strength. At 0.5 M NaCl, the viscosity feature observed for the paste without dispersant (Figure 6) was hindered at low ionic strength, but for the LMW fraction at 0.5 M NaCl, it reoccurred. The LMW fraction did not exhibit increased adsorption at high ionic strength (Figure 3). This suggested that the pastes prepared with the LMW fraction at 0.5 M NaCl maintained similar surface coverage to that at low ionic strength. However, the reduced Debye length at higher ionic strength may lead to reduced dispersion performance and increased viscosity. Viscosity was generally lower for the HMW fraction, indicating a more salt-tolerant fraction, likely due to increased steric hindrance in the ionic pastes.

##### Effect of Ionic Composition on Rheology of the Pastes

Storage modulus and loss factor in the linear viscoelastic region and yield stress for the pastes with monovalent ions or a mixture of monovalent and divalent ions can be found in Table 2.

When no dispersant was present, no change in G′ was observed, and no LVR was present within the measured strain region. For pastes stabilized by lignosulfonate, the presence of divalent ions resulted in increased G′ at 0.1 M and 0.5 M ionic strength, indicating stronger interparticle network formation, likely due to enhanced double-layer compression and cation bridging. Generally, the presence of divalent ions resulted in an increase in loss factor, and the LVR was seen to shrink with the addition of divalent ions. Reduced LVR results in a mechanically stronger but less deformation-tolerant paste when divalent ions are present.

At 0.1 M ionic strength, the yield stress for the fractions (LMW and HMW) did not change significantly with the addition of divalent ions. At 0.5 M ionic strength, however, an increase was seen for all dispersed pastes. The most drastic increase was for the HMW fraction, where the yield stress with monovalent electrolyte was 4.70 Pa, while in the mixed ionic solution, it was 25.40 Pa. Increased yield stress indicates stronger TiO_2_-TiO_2_ interactions, and a higher force is needed to induce flow.

**Figure 8:**
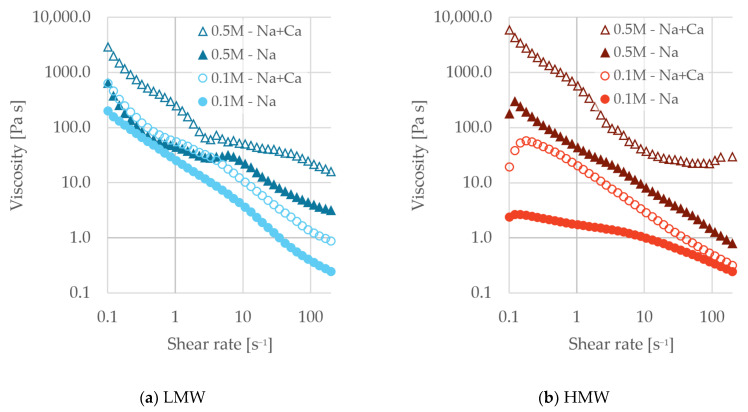
Viscosity as a function of shear rate for the pastes at ionic strengths of 0.1 M (circles) and 0.5 M (triangles) with only monovalent (Na, filled) or a mixture of monovalent and divalent ions (Na + Ca, unfilled). The pastes were prepared either with LMW (**a**) or HMW (**b**) lignosulfonate fractions.

This was also reflected in the flow curves, where increased viscosity was observed with increased ionic strength and calcium content, as shown in Figure 8. The viscosity feature observed in the pastes without dispersant at 10 s^−1^ (Figure 6) was absent under low ionic strength for all samples. However, in highly saline systems containing divalent ions, this feature reappeared across all lignosulfonate fractions. This suggested a greater degree of interparticle interactions.

Previously, it has been shown that the HMW fraction is the most tolerant to changes in ionic strength. From the amplitude sweep and flow curve, it is evident that the LMW is the most tolerant to changes in ionic composition, specifically the addition of divalent ions. Divalent ions are more effective in screening charges, so it is likely that lignosulfonate molecules adopt a more compact conformation. According to previous work [31], the size of the HMW fraction shows a larger dependence on ionic conditions. Although the HMW fraction showed the lowest viscosity under the mild conditions, under strongly saline, Ca^2+^-rich conditions at the current dosage, its dispersing effect was markedly reduced.

## 4. Conclusions

In suspensions containing hydrophilic particles (TiO_2_), lignosulfonate can reduce particle size, viscosity, and network formation. In the absence of ions, no adsorption occurs, but stabilization is attributed to depletion stabilization by non-adsorbing lignosulfonates, reflected by low particle size and low viscosity. With the increase in ionic strength, adsorption increases, leading to a more interconnected particle network with higher viscosity. Divalent ions promote increased adsorption and higher viscosity due to divalent cation bridging.

Both low- and high-molecular-weight lignosulfonates exhibit dispersing effects, with the high-molecular-weight polymer providing the lowest viscosity under low to moderate ionic strengths. At high ionic strength with divalent ions, however, adsorption combined with chain compaction and Ca^2+^ bridging leads to increased viscosity, likely due to bridging effects. The low-molecular-weight lignosulfonate yields a smaller viscosity reduction but shows less variation in adsorption behavior with electrolyte composition.

It has been shown that the combination of adsorption measurements with particle size determination and rheological investigations can provide a more complete view of effects such as depletion, adsorption, and Ca^2+^ bridging. The results indicate that it is important for formulators to consider the molecular weight of the lignosulfonate depending on the ionic conditions of their application. For low and intermediate ionic strength, the high-molecular-weight fraction showed the best performance, while for high salt systems containing divalent ions, the low-molecular-weight fraction was more suitable. Formulations may also benefit from the determination of dispersant dosage under the relevant operating conditions to ensure surface coverage and performance. These findings are directly relevant to industrial applications involving mineral-rich suspensions and pastes, such as coatings, construction materials, and process streams with variable electrolyte composition.

These findings can support formulation strategies in systems with variable ionic environments, such as mineral-rich suspensions or industrial effluents. In industrial systems, the ion composition may be more complex, and subsequent research should investigate the effects of hard water and other electrolyte compositions. The dispersion mechanism in salt-free systems was attributed to depletion stabilization, but further work is required to confirm and refine the understanding of the lignosulfonate dispersion mechanism in salt-free systems. Further work could investigate long-term stability and temperature effects to extend applicability. In addition, the behavior of lignosulfonates on hydrophobic particle surfaces could be examined to complement the present findings. To highlight the competitiveness of the results, a comparison with similar materials should be made. This may include fractionated lignosulfonate from other sources, other bio-based dispersants with low carbon footprints, and synthetic dispersants.

## Figures and Tables

**Table 1:** Storage modulus and loss factor in the linear viscoelastic region and the yield stress for the pastes at different ionic strengths.

		0 M	0.1 M	0.5 M
Storage modulus	No LS *	148,195	211,630	180,090
[Pa]	SM	170	1530	45,378
	LMW	1223	19,564	153,533
	HMW	13	62	7671
Loss factor	No LS *	0.43	0.45	0.50
[Pa]	SM	0.10	0.12	0.36
	LMW	0.08	0.13	0.22
	HMW	0.55	0.11	0.19
Yield stress	No LS	N/A **	N/A **	N/A **
[Pa]	SM	1.97	3.11	1.96
	LMW	4.20	11.09	4.13
	HMW	0.15	8.25	4.70

* Storage modulus and loss factor are given at the lowest measured strain. ** No yield stress within the measured strain range.

**Table 2:** Storage modulus and loss factor in the linear viscoelastic region and yield stress for the pastes with monovalent salt or a mixture of monovalent and divalent salt. The pastes are either without dispersant (no LS) or stabilized by SM, LMW, or HMW fractions.

		0.1 M		0.5 M	
		Mono.	Mixed.	Mono.	Mixed.
Storage modulus	No LS *	211,630	217,395	180,335	157,505
[Pa]	SM	1530	24,982	45,378	447,800
	LMW	19,564	132,308	153,533	794,350
	HMW	62	1004	7671	999,845
Loss factor	No LS *	0.45	0.43	0.50	0.34
[Pa]	SM	0.12	0.27	0.36	0.38
	LMW	0.13	0.20	0.22	0.59
	HMW	0.11	0.12	0.19	0.32
Yield stress	No LS	N/A **	N/A **	N/A **	N/A **
[Pa]	SM	3.11	8.74	1.96	9.19
	LMW	11.09	9.83	4.13	9.56
	HMW	8.25	8.57	4.70	25.40

* Storage modulus and loss factor are given at the lowest measured strain. ** No yield stress within the measured strain range.

## Data Availability

The raw data supporting the conclusions of this article will be made available by the authors on request.

## Supplementary Materials

The following supporting information can be downloaded at https://www.mdpi.com/article/10.3390/polym18020270/s1, Figure S1: Ultrafiltration scheme; Figure S2: Pictures of TiO_2_ pastes; Figure S3: QCM-D data handling; Figure S4: Zeta potential; Table S1: Ionic composition of Norwegian groundwater; Table S2: Replicates for rheology.

## Abbreviations

The following abbreviations are used in this manuscript:

Đ	Dispersity (Mw/Mn)
DLVO	Derjaguin–Landau–Verwey–Overbeek theory
G′	Storage modulus
G″	Loss modulus
HMW	High-molecular-weight fraction lignosulfonate
IEP	Isoelectric point
LMW	Low-molecular-weight fraction lignosulfonate
LS	Lignosulfonate
LVR	Linear viscoelastic range
QCM-D	Quartz crystal microbalance with dissipation monitoring
SM	Start material
