# Supplementary material for: Dispersion Mechanisms of Lignosulfonates in Concentrated TiO2 Suspensions and Pastes: The Effects of Molecular Weight Distributions and Ionic Composition
Source: Polymers (Basel). 2026 Jan 20;18(2):270. doi: 10.3390/polym18020270 (PMC12846172; doi:10.3390/polym18020270)
Supplement: Supplementary file 1 [file polymers-18-00270-s001.zip › polymers-4073942-supplementary.pdf]

Supporting Information

Dispersion Mechanisms of Lignosulfonates in Concentrated TiO_2 Suspensions and Pastes: The Effects of Molecular Weight Distributions and Ionic Composition

Veslemøy Margrethe Selvik [1,2], Carlos Salas-Bringas[1] and Gisle Øye[2,*]

1 Borregaard ASA, 1701 Sarpsborg, Norway; veslemoy.selvik@borregaard.com (V.M.S.) carlos.salas.bringas@borregaard.com (C.S-B.)
2 Ugelstad Laboratory, Department of Chemical Engineering, Norwegian University of Science and Technology (NTNU), 7491 Trondheim, Norway
* Correspondence: gisle.oye@ntnu.no

S1. Ultrafiltration scheme

Fractionation on molecular weight was achieved through ultrafiltration. A new sample of the start material was used in the preparation for each fraction. The low molecular weight fraction (LMW) was prepared by filtrating through a membrane with 1 kDa molecular weight cut off. The retentate was then used as feed for a second filtration through a 5 kDa membrane where the permeate was collected. For the high molecular weight (HMW) fraction a single filtration through a 300 kDa membrane was carried out and the retentate used as the fraction. Figure S1 illustrate the ultrafiltration scheme.

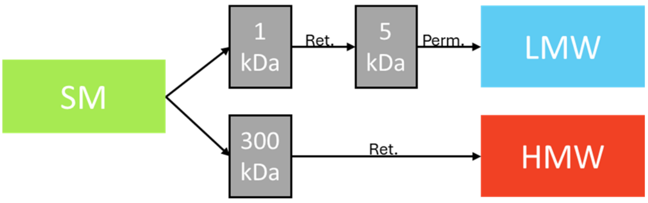

Figure S1. Ultrafiltration scheme for the preparation of a low molecular weight (LMW) and high molecular weight (HMW) fraction from the start material (SM). Molecular weight cut off of the membranes are indicated.

S2. Ionic composition

To determine the ionic composition used in the study the divalent and monovalent ions present in Norwegian ground water was chosen as a basis. Data was collected in November 2024 from Norwegian municipalities reporting of water quality. Monovalent ions ($^{+}$) were primarily Na^{+} and K^{+}, while divalent ions ($^{2+}$) were primarily Ca^{2+} and Mg^{2+}. Concentration of ions with different valency, as well as the ratio between them is shown in **Table S1**. A mean molar ratio of 3:1 Ca^{2+}:Na^{+} was found.

Table S1. Ionic composition from Norwegian ground water. The table indicates the sampling location, year of analysis, concentration of monovalent ions (C_{+} ,primarily Na^{+} and K^{+}) and divalent ions (C_{2+}, primarily Ca^{2+} and Mg^{2+}), as well as ratio of divalent to monovalent ions. The mean ratio is 2.9 C_{2+}:C_{+}.

Municipality	Location	Year	C_+ [mol/L]	C_{2+} [mol/L]	$C_{2+}: C_+$ [-]	Source See list below
**Trondheim**	*Jonsvatnet*	*2023*	1.70E-04	6.21E-04	3.7	**a**
	Jonsvatnet	*2022*	1.71E-04	5.82E-04	3.4	
	Jonsvatnet	*2021*	1.71E-04	5.37E-04	3.1	
**Oslo**	*Average value*	*2021*	8.55E-05	4.21E-04	4.9	**b**
**Bergen**	*Espeland*	*2022*	1.09E-04	4.19E-04	3.8	**c**
	Jordalsvatnet	*2022*	2.48E-04	4.64E-04	1.9	
	Kismul	*2022*	3.44E-04	3.84E-04	1.1	
	Risnes	*2022*	8.70E-05	3.47E-04	4.0	
	Svartediket	*2022*	1.57E-04	4.72E-04	3.0	
	Risnes	*2022*	3.00E-04	3.74E-04	1.2	
**Kristiansand**	*Rossevann*	*2023*	2.91E-04	4.74E-04	1.6	**d**
	Tronstad	*2023*	2.04E-04	5.99E-04	2.9	
**Mean**					**2.9**	

Sources:

a) https://www.trondheim.kommune.no/tema/veg-vann-og-avlop/vann-og-avlop/om-vann-og-avlop/drikkevannskvalitet/
b) https://www.oslo.kommune.no/vann-og-avlop/drikkevannskvalitet/#gref
c) https://www.bergen.kommune.no/innbyggerhjelpen/vann-vei-og-trafikk/vann-og-avlop/vann-og-vannforsyning/drikkevannskvalitet
d) https://www.kristiansand.kommune.no/navigasjon/bolig-kart-og-eiendom/vann-og-avlop/vannforsyning/

S3. Determination of solid loading for concentrated TiO_2 suspensions

Concentrated TiO_2 suspensions with solid loading ranging from 70% to 80% are shown in **Figure S2**.

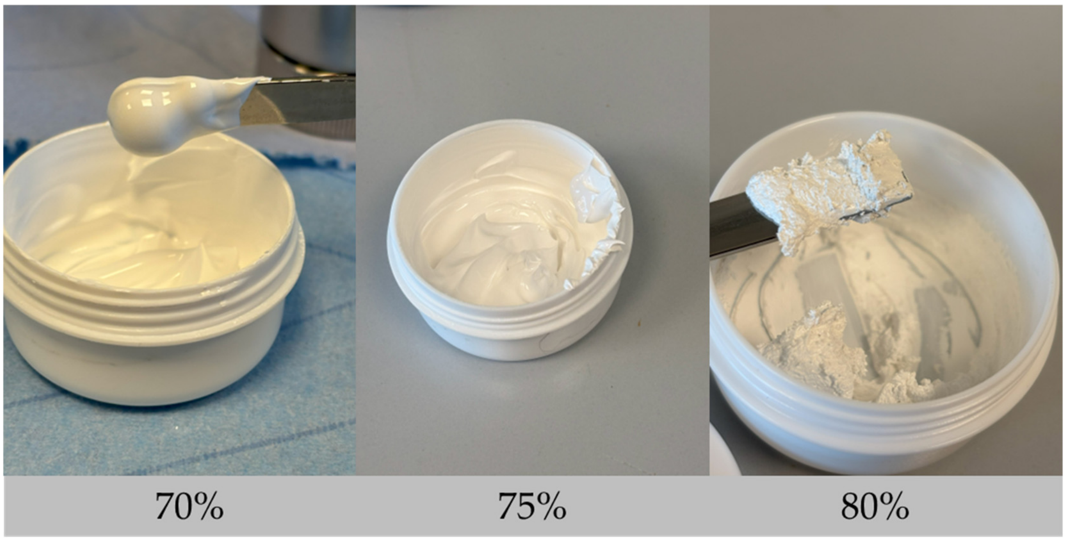

Figure S2. Images showing the texture of concentrated suspensions with 70 wt%, 75 wt% and 80 wt% solid loads.

S4. Surface area

BET (Brunauer-Emmett-Teller) analysis was used to measure surface area of TiO_2 particles. Nitrogen adsorption measurements were performed using a TriStar II Plus surface area and porosity analyzer (Micromeritics, USA). The sample was degassed at 90°C

for 1 hour then 350°C for 4 hours and left overnight before being measured at liquid nitrogen temperature. Three replicates were made, and the surface area was determined to be 15.55±0.08 $m^2\,g^{-1}$.

Average particle size was calculated based on the measured BET surface area (S) and density (ρ) from the manufacturer. The particles are assumed to be spherical and monodisperse. The calculated size must therefore be considered an average value.

$$d = \frac{6}{\rho \cdot S} = \frac{6}{750\ kg/m^3 \cdot 15550\ m^2/kg} = 514\ nm$$

S5. QCM-D data handling

Figure S3 shows an example of the data handling used. **Figure S3**(**a**) shows a representative example of the frequency shift normalized with the harmonic number ($\Delta f/n$) and dissipation (D) as a function of time. A moving average has been applied to eliminate noise. The start of the adsorption and desorption stages are indicated by vertical lines. **Figure S3**(**b**) shows dissipation as a function of frequency shift. The line $D = -0.1\Delta f/n$ is plotted for reference as a slope below 0.1 indicates that the Sauerbrey model is valid. The adsorbed mass calculated according to the Sauerbrey equation using the 3rd overtone is shown in **Figure S3**(**c**). The reported mass in this publication is the mean of the last 10 minutes of in the desorption stage and is indicated by a circle in the figure.

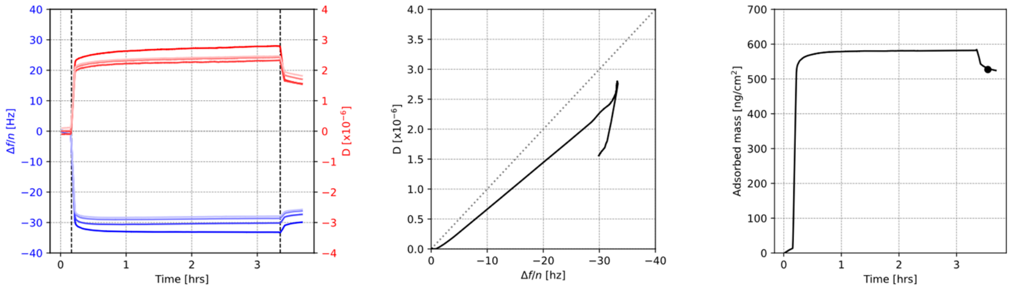

(**a**) Dissipation (***D***) and frequency shift (**Δ*f***, Hz).

(**b**) Dissipation (***D***) plotted as function of frequency shift (**Δ*f***) for the 3rd overtone.

(**c**) Areal mass (ng/cm²) calculated by Sauerbrey equation.

Figure S3. Dissipation (***D***, 10^{-6}), frequency shift (**Δ*f*/*n***, Hz) and areal mass (ng/cm²) shown to illustrate the data handling of QCM-D results. In Figure (**a**) vertical lines show the transition between the baseline, adsorption and desorption stages. In Figure (**b**) a line with slope 0.1 is shown to for reference as this describes the boundary for the Sauerbrey model. In Figure (**c**) the reported equilibrium mass is indicated by a circle.

S6. Reproducibility of rheology

Reproducibility of rheological behavior was assessed by performing replicate experiments at the center points of the experimental design, as recommended by DOE framework. The results from these center point replicates showed good agreement, indicating that the experimental procedures are robust and that the observed effects are reproducible under the tested conditions. From these preliminary studies it is evident that the largest variability occurs for the high molecular weight (HMW) fraction. **Table S2** summarize the viscosity at three different shear rates (1 s^{-1}, 10 s^{-1}, and 100 s^{-1}) for each sample. The mean and standard deviation for each sample at each shear rate is shown as well as the percentage error.

Table S2. Viscosity measured at three shear rates (1 s^{-1}, 10 s^{-1}, and 100 s^{-1}) for concentrated TiO_2 suspensions dispersed by lignosulfonate. The dispersants used were either unfractionated start material (SM), low molecular weight (LMW), or high molecular weight (HMW) lignosulfonate. Reported values represent the mean of three replicates, along with standard deviation and percentage error.

		Viscosity [Pa s]			Viscosity [Pa s]		
		**Rep 1**	**Rep 2**	**Rep 3**	**Mean**	**St.dev.**	**%**
**SM**	**1 s^{-1}**	1.04E+02	9.00E+01	1.03E+02	9.89E+01	6.35E+00	6 %
	**10 s^{-1}**	3.88E+01	3.74E+01	3.91E+01	3.84E+01	7.54E-01	2 %
	**100 s^{-1}**	1.12E+01	1.01E+01	1.11E+01	1.08E+01	4.84E-01	4 %
**LMW**	**0.1 s^{-1}**	1.29E+02	1.29E+02	9.02E+01	1.16E+02	1.84E+01	16 %
	**10 s^{-1}**	4.16E+01	4.30E+01	3.80E+01	4.09E+01	2.14E+00	5 %
	**100 s^{-1}**	1.29E+01	1.54E+01	9.81E+00	1.27E+01	2.27E+00	18 %
**HMW**	**0.1 s^{-1}**	8.51E+01	2.41E+02	2.29E+02	1.85E+02	7.07E+01	38 %
	**10 s^{-1}**	3.05E+01	4.47E+01	4.10E+01	3.87E+01	5.99E+00	15 %
	**100 s^{-1}**	1.02E+01	1.91E+01	1.70E+01	1.54E+01	3.81E+00	25 %

S7. Zeta potential of TiO_2 particles

Zeta potential measurements of bare TiO_2 particles in UPW were conducted using a Zetasizer Nano ZS (Malvern, UK) with a 3-minute equilibration period. 0.1 wt% TiO_2 was dispersed in water and diluted 500x to reduce opacity, and pH was adjusted using HCl and NaOH. The Hückel approximation was used to correlate electrophoretic mobility and zeta potential. The zeta potential of TiO_2 particles measured for a range of pH is shown in **Figure S4**. A sigmoid function was fitted to the data, and the IEP was found to be 4.6.

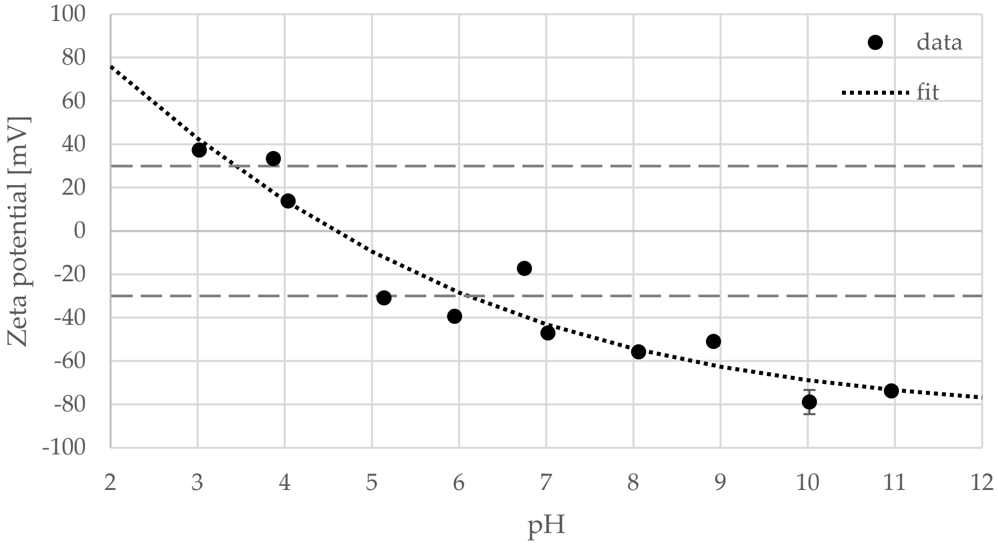

Figure S4. Zeta potential of TiO_2 for a range of pH. A sigmoid fit is plotted and the boundaries for stable suspensions are outlined. Standard deviation from experimental parallels is included as error bars. R^2 value of the fit is 0.91.
